# Risk-Stratified Venous Thromboembolism Chemoprophylaxis After Total Joint Arthroplasty: Evaluation of an Institutional Approach

**DOI:** 10.3390/jcm14020366

**Published:** 2025-01-09

**Authors:** Sara J. Hyland, Maria J. Fada, Michelle Secic, Robert A. Fada, Marie M. Lockhart, Richard H. Parrish

**Affiliations:** 1 Department of Pharmacy, OhioHealth Grant Medical Center, Columbus, OH 43215, USA; 2 Ohio University Heritage College of Osteopathic Medicine, Athens, OH 45701, USA; 3 Secic Statistical Consulting, Inc., Cleveland, OH 44106, USA; 4 Department of Orthopedics, OhioHealth Grant Medical Center, Columbus, OH 43215, USA; 5 OhioHealth Research Institute, Columbus, OH 43214, USA; 6 Mercer University School of Medicine, Columbus, GA 31901, USA

**Keywords:** aspirin, chemoprophylaxis, postoperative complications, postoperative hemorrhage, risk stratification, total joint arthroplasty, venous thromboembolism

## Abstract

**Background/Objectives**: The optimal venous thromboembolism (VTE) chemoprophylaxis approach after hip or knee total joint arthroplasty (TJA) remains controversial. This study aimed to characterize antithrombotic-related complications associated with various chemoprophylaxis regimens after TJA and to assess our current institutional risk-stratified prescribing tool. **Methods**: This retrospective case–control study and regression analysis included elective unilateral TJA patients at a single institution between 1 July 2015 and 31 December 2021. The primary outcome was a composite of antithrombotic-related complications within 30 days of surgery, including thrombotic and hemorrhagic/wound-related adverse events. The duration of anticoagulant chemoprophylaxis prescribed prior to aspirin monotherapy (0–28 days) was compared between patients who did vs. did not experience a complication, with stratification by institutionally defined VTE risk categories (Routine, Moderate, or High Risk). The complication rate was then assessed as a function of anticoagulant duration within each risk subgroup. **Results**: The study included 5420 patients, with 279 (5.2%) experiencing ≥1 complication. Routine VTE risk patients experienced few complications, with no significant difference between aspirin monotherapy and various initial anticoagulant durations (*p* = 0.6118). Moderate and High VTE Risk patients saw significantly lower complication rates with initial anticoagulant prophylaxis of increasing durations (*p* = 0.0090 and *p* = 0.0050), with a significant overall effect of VTE Risk strata observed (*p* = 0.0006). **Conclusions**: When both bleeding and thrombotic events are considered, anticoagulant-to-aspirin regimens were associated with lower complication rates than aspirin monotherapy in higher risk patients, while routine patients saw no significant benefit over aspirin. Our risk-stratified prescribing approach should be prospectively evaluated.

## 1. Introduction

Preoperative risk assessment for venous thromboembolism (VTE) and hemorrhage began in the 1980s and led to the development of scoring systems to stratify surgical patients and guide prophylactic measures. Prevention of VTE after total joint arthroplasty (TJA) of the hip or knee became an integral component of successful surgical care. However, perioperative practice has substantially evolved since previous VTE prevention guidelines, specific to joint replacement, were published over a decade ago [1,2]. Bleeding events now outpace thromboses as the predominant postoperative complications, disrupting prior assumptions of the relative benefits and risks of chemoprophylaxis regimens [3,4,5]. Recent randomized controlled trials have challenged the need for full courses of anticoagulant chemoprophylaxis after TJA as compared to aspirin in the modern era of enhanced recovery pathways and multimodal risk reduction strategies [6,7,8,9]. Multiple risk-stratified protocols for VTE prevention after TJA have also accumulated, suggesting that prophylaxis strategies can be tailored to patient-specific factors to allow for sustained efficacy, with less exposure to higher-risk and/or expensive anticoagulants [10,11,12,13,14,15,16]. Still, the ideal approach to postoperative chemoprophylaxis that limits both thromboembolic and hemorrhagic complications for various patient risk strata remains elusive [5].

Available prediction models for postoperative VTE after TJA suffer from a variety of limitations, including lack of point-of-care practicality, low generalizability to modern practice, under-representation of higher risk patients, lack of robust validation data, absent/insufficient consideration for bleeding risk, and/or lack of validated prescribing guidance based on risk [5,13,15,17]. Hence, no VTE risk stratification in orthopedic surgery to date has been sufficiently externally validated to support recommendations for widespread utilization, and further development of risk-stratified chemoprophylaxis approaches is needed [5,16]. Specifically, there exists a persistent need to determine which TJA patients benefit from an anticoagulant or hybrid anticoagulant-to-aspirin chemoprophylaxis approach over aspirin monotherapy, and the optimal duration of anticoagulant that should be considered before de-escalation to aspirin in higher risk patients [16].

At our hospital-based orthopedic surgery center, risk-stratified VTE chemoprophylaxis prescribing guidance has been in place since 2016 and iteratively refined based on new literature, though prophylaxis regimens were never standardized. This practice has yielded a real-world database of elective TJA patients of diverse VTE risks who received a variety of chemoprophylaxis regimens in a modern enhanced recovery care model. This study aimed to evaluate antithrombotic-related complications associated with VTE risk-stratified chemoprophylaxis regimens to help elucidate an optimal strategy that limits both thrombotic and hemorrhagic adverse events for patients of different VTE risk strata. We also sought to explore the value of our current institutional VTE risk stratification scheme in discriminating patients at greater risk for complications and in guiding VTE chemoprophylaxis prescribing to minimize adverse events.

## 2. Materials and Methods

### 2.1. Study Population and Setting

We pursued a retrospective, single-center, case–control study with regression analysis after garnering institutional review board approval. We adhered to the Strengthening the Reporting of Observational Studies in Epidemiology (STROBE) reporting guidelines in preparing our manuscript [18]. Adult patients undergoing an elective, unilateral, primary or revision TJA at our institution from 1 July 2015 to 21 December 2021 were assessed for inclusion if they were prescribed VTE chemoprophylaxis with an anticoagulant and/or aspirin in the immediate postoperative period. Those undergoing surgery for fracture/trauma or oncologic indications were not eligible for inclusion. Patients who underwent multiple TJAs throughout the study timeframe were eligible for study inclusion at each point of elective TJA. Patients were excluded if they were prescribed a therapeutic anticoagulant medication prior to admission, ordered new therapeutic anticoagulation postoperatively for a non-thrombotic indication (e.g., heparin infusion for postoperative atrial fibrillation), or prescribed postoperative VTE chemoprophylaxis agents other than aspirin, apixaban, or enoxaparin.

The study institution is an orthopedic surgery center within a 640-bed community teaching hospital where 900–1000 total hip and knee arthroplasty procedures are performed annually. Near-universal use of regional and neuraxial anesthesia techniques for elective TJA was implemented previously. Tranexamic acid use was protocolized early in the study timeframe to 1000–2000 mg administered intravenously in addition to 1000 mg administered via intraoperative irrigation. Standardized institutional nonpharmacologic VTE prophylaxis practices have long included compression stockings and pneumatic compression devices applied intraoperatively and continued during admission. All patients are mobilized on the day of surgery and twice daily during admission by physical therapists with expertise in TJA rehabilitation, unless patient-specific circumstances preclude mobilization. The average post-TJA hospital length of stay (LOS) steadily decreased throughout the study timeframe from 2.86 days in 2015 to 1.7 days in 2021, likely owing to interprofessional continuous quality improvement efforts for TJA care at our institution.

### 2.2. Study Objectives, Groups, and Outcome Variables

**Figure 1:**
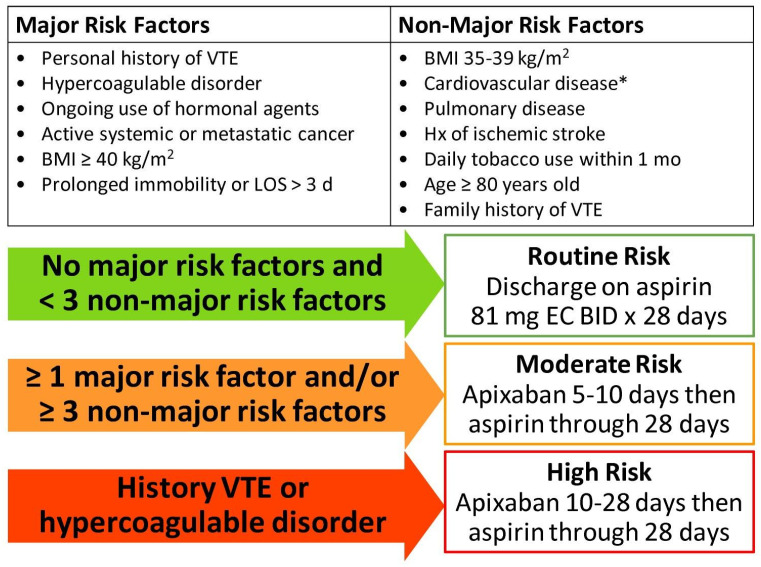
The GRANT prescribing tool (The Grant Medical Center Risk Assessment for Negating Thromboses). Legend: this summarizes our current institutional risk-stratified recommendations for VTE chemoprophylaxis after elective total hip or knee arthroplasty. The orthopedic service pharmacist provides patient-specific recommendations for discharge using this evidence-based institutional guidance, though the ultimate prescription is at the surgeon’s discretion. Note: all patients are recommended apixaban during hospitalization at least until adequate postoperative mobility is achieved, barring significant bleeding concerns. *: Diabetes, hypertension, and hyperlipidemia are not counted as risk factors individually or in combination. Abbreviations: BID—twice daily; BMI—body mass index; EC—enteric coated; Hx—personal history; LOS—length of stay; VTE—venous thromboembolism.

The first objective of this study was to characterize antithrombotic-related complications associated with risk-stratified VTE chemoprophylaxis regimens after TJA, including both thrombotic and hemorrhagic adverse events, in order to propose optimal prescribing strategies for patients of different VTE risk strata. Secondarily, we sought to retrospectively assess our current institutional VTE risk-stratified chemoprophylaxis prescribing guidance (Figure 1). A case–control design was utilized whereby eligible patients were assigned to study groups based on whether or not they experienced an antithrombotic-related complication within 30 days of surgery. A logistic regression model was planned to control for known patient-specific factors that constitute confounding variables and determine predictive characteristics to include in an optimal risk stratification scheme. Then, patients were sub-grouped into institutionally defined Routine, Moderate, or High VTE Risk strata and the antithrombotic-related complication rate was assessed as a function of anticoagulant duration utilized (0–28 days) prior to aspirin.

The primary outcome was a composite of antithrombotic-related complications occurring within 30 days postoperatively, which included both clinically significant thrombotic and hemorrhagic/wound-related adverse events (Table 1). This composite was chosen instead of VTE rate alone because both serious bleeding and thrombotic events should be assessed in TJA risk stratifications to provide a more inclusive measure of risks and benefits of a given chemoprophylaxis strategy [5]. Isolated distal DVT and subsegmental PE were excluded due to their waning clinical significance in current clinical practice guidelines [19]. Death and deep surgical site infection were not discretely included in the composite primary outcome to limit Type I attribution error in adjudicating these as antithrombotic-related complications, though patients who experienced any of the composite complication events before expiring or developing prosthetic joint infections were included and adjudicated as having experienced the primary outcome. Postoperative complications were assessed through multiple mechanisms, including a manual review of all readmissions and reoperations for applicable diagnoses, a manual review of quality department complications case reviews, and electronic data query via diagnoses codes as provided in the Supplementary Material. Demographic variables and procedure data, including hospital LOS, were also collected via electronic data reporting and manual chart review. All variables assessed were data routinely collected during the course of clinical care and documented in the electronic medical record, hence no plans for missing data were deemed indicated.

### 2.3. VTE Chemoprophylaxis Prescribing Practices During Study Time Frame and Current Institutional Risk Stratification Development

Our longtime institutional practice and preference has been to prescribe aspirin for VTE chemoprophylaxis at hospital discharge for four weeks’ duration, due to its favorable effectiveness, safety, and cost [20]. A series of postoperative thrombotic complications in high-risk TJA patients led to a practice shift toward a more risk-stratified chemoprophylaxis approach in 2016, whereby higher risk patients were recommended to receive various durations of initial anticoagulant chemoprophylaxis prior to aspirin for the remaining course. This hybrid anticoagulant-to-aspirin chemoprophylaxis approach capitalizes on the front-loaded distribution of postoperative VTE risk to spare excess anticoagulant exposure, and has been validated in randomized controlled trials [8,12,21,22,23]. Though risk-stratified guidance suggesting a hybrid chemoprophylaxis approach in higher risk patients was provided, prescribing practices were never protocolized and remained at the surgeons’ discretion. This evolution and non-standardization of institutional practice has yielded a real-world database of TJA patients of diverse VTE risk strata who were prescribed a variety of chemoprophylaxis regimens during the study timeframe, including aspirin monotherapy or various durations of anticoagulant prior to aspirin for the remaining four-week course. Considering institutional case volume, we felt this database would yield sufficient population size and variety so as to supply adequate counterfactual construction for a retrospective evaluation of our current risk stratification scheme.

Our current institutional guidance on risk-stratified VTE chemoprophylaxis after elective TJA is summarized in Figure 1 and was refined after the present study timeframe in 2022 utilizing evidence-based risk factors most pertinent to modern enhanced recovery practice [21,24,25,26]. Risk factors with statistically significant odds ratios, ≥1.8, in prior studies were generally considered for major risk factors when deriving our current stratification scheme, while statistically significant risk factors with lesser odds ratios were generally classified as non-major. A patient’s unique combination of risk factors are integrated into a 3-tiered stratification to account for the effect of total comorbidity burden in conferring VTE risk [13,25]. Previous iterations of VTE chemoprophylaxis prescribing guidance in place during the study timeframe utilized somewhat different risk factors and anticoagulant agents based on available literature at the time. Hence, no providers had access to the current risk stratification scheme while practicing during the study timeframe, allowing for retrospective exploration of the current scheme’s discriminating value and of the expected relationship between complication rates and anticoagulant duration for currently defined risk strata.

The preferred anticoagulant chemoprophylaxis agent changed from enoxaparin to apixaban in 2018, based on evidence suggesting comparable efficacy, lower bleeding risk, and less burden of administration [27,28]. All patients were recommended to receive apixaban 2.5 mg orally twice daily (previously enoxaparin 40 mg subcutaneously once daily) during the postoperative hospital stay, starting 8–24 h after anesthesia end time, at least until adequate postoperative mobility was achieved. Aspirin dosing was usually enteric-coated 81 mg twice daily. The ultimate VTE prophylaxis regimen was determined by the surgeon after collaborative assessment during postoperative multidisciplinary patient rounds. The orthopedic service pharmacist then assessed for medication adherence or affordability barriers to the prescribed regimen and provided individualized patient education prior to discharge [29]. Post-discharge, patient adherence to prescribed medications was generally assessed and reiterated via a phone call from an orthopedic unit nurse conducted 1–3 days after hospital discharge and at surgeon office follow-up 2–3 weeks after discharge, though patient medication compliance was not formally tracked in the medical record.

### 2.4. Data Analyses

All study data were summarized with descriptive statistics and separately for those with vs. without the antithrombotic-related complication composite primary outcome. Pre-specified subgroup analyses were pursued by patient VTE risk strata and by anticoagulant agent. In the regression analyses, continuous variables were compared between groups using *t*-tests or Wilcoxon rank sum tests, as appropriate. Categorical variables were compared between groups using chi-square tests or Fisher’s exact tests, as appropriate. From these univariable tests, a pool of potential risk factors were identified. Multivariable logistic regression was used to determine an optimally predictive model for the primary outcome. The duration of initial anticoagulant prophylaxis was assessed univariably to obtain an assessment of the unadjusted effect on the primary outcome. The univariably significant factors were used in multivariable modeling to identify an optimal set of risk factors for the primary outcome. Multivariable logistic regression methods were then used, including assessment of multicollinearity and then backward elimination for model-building. This allowed for an assessment of the composite complication primary outcome that was adjusted for baseline risk factors. No corrections for multiple comparisons were planned. Results with a *p*-value ≤ 0.05 were considered statistically significant. All analyses were completed in SAS, v9.1. 

The study timeframe was chosen to allow for the largest possible dataset from our institution’s current electronic medical record system, and was estimated to provide approximately 5000 patients who would meet the study criteria for inclusion based on our average annual TJA procedure rate. With a maximum of 5000 patients, and a conjectured composite complication rate between 2% and 6%, we expected a minimum number of composite complication events of approximately 100. Given the general recommendation for logistic regression is at least 10 events for every additional factor in the final model, our final model could have up to 10 statistically significant risk factors [30].

In the secondary analyses, the composite antithrombotic-related complication rate was assessed as a function of anticoagulant duration (0–28 days) prior to aspirin within each institutionally defined VTE risk subgroup via chi-square tests. An overall Cochran–Mantel–Haenszel test was used to assess the influence of VTE risk stratum on the complication-anticoagulant duration relationship. These secondary analyses were exploratory and should be considered hypothesis-generating in nature.

## 3. Results

**Figure 2:**
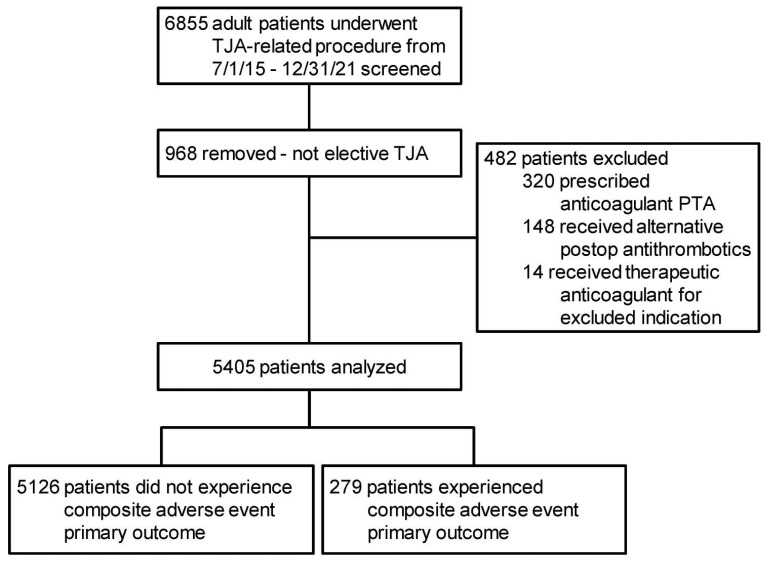
Population determination. Legend: PTA—prior to admission; TJA—total joint arthroplasty (of the hip or knee).

A total of 5405 patients met study criteria and were analyzed, 279 (5.2%) of whom experienced an antithrombotic-related complication (Figure 2). Bleeding/wound complications were more frequent than thrombotic complications (Table 2).

**Figure 3:**
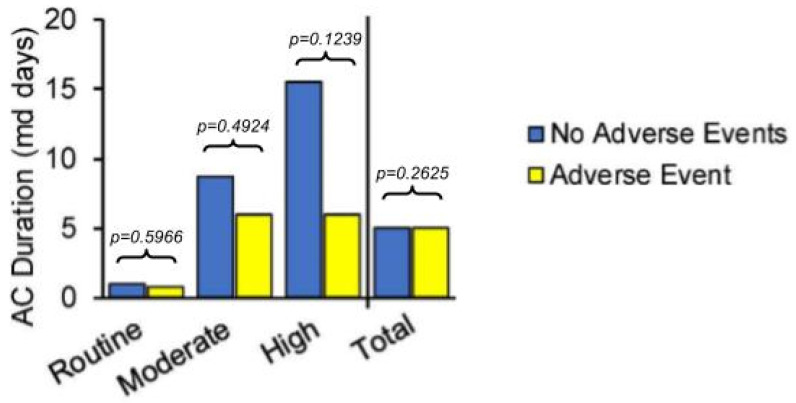
Case–control analysis of composite complication primary outcome by VTE risk strata subgroups and in total population. Legend: the population that did not experience a complication (“No Adverse Event” group) is represented by blue bars and the complication population (“Adverse Event” group) is represented by yellow bars. The median duration of anticoagulant did not differ between Adverse Event and No Adverse Events groups at the population level (5 days vs. 5 days, *p* = 0.2625) or in the Routine VTE Risk subgroup (0.75 day vs. 1 day, *p* = 0.5966, n = 1084). Non-significant differences were seen in the Moderate VTE Risk subgroup (6 days vs. 8.75 days, *p* = 0.4924, n = 3993) and High VTE Risk subgroup (6 days vs. 15.5 days, *p* = 0.1239, n = 327). The population was truncated to those with anticoagulant durations of ≤30 days for these subgroup analyses to align with the complication assessment timeframe and avoid immortal time bias. Abbreviations: AC—anticoagulant; md—median.

The duration of anticoagulant prophylaxis prior to aspirin was not found to be a significant predictor of the primary outcome at the population level (median 5 days vs. 5 days, *p* = 0.2625), though non-significant differences were observed across the VTE risk strata subgroups (Figure 3).

Our institutionally defined VTE risk strata were significantly differentially distributed between the complication and no-complication groups (*p* < 0.001), with the complication group seeing greater proportions of Moderate and High-risk patients than the no-complication group (Table 3). Additionally, a significant differential distribution of the primary outcome was observed across the VTE chemoprophylaxis exposure subgroups (*p* = 0.003) such that patients who experienced an antithrombotic-related complication were more likely to have received enoxaparin than the total population (70.3% vs. 59.3%) (Table 3).

**Figure 4:**
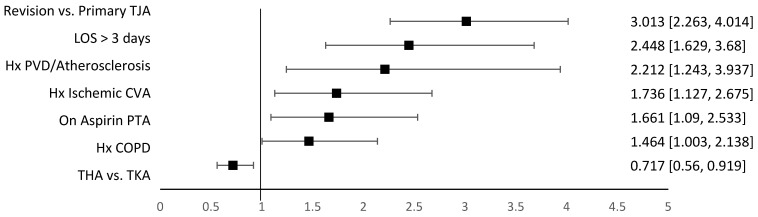
Multivariable logistic regression analysis for predictors of antithrombotic-related complications’ composite primary outcome. Footnote: after adjusting for multicollinearity, the final multivariable logistic regression model retained seven variables as being significant predictors of the composite complication primary outcome. Variables are ordered by odds ratio. Bolded *p*-values indicate statistically significant findings at prespecified alpha = 0.05. Since a personal history of VTE was not found to be a significant predictor of the composite complication primary outcome in our model but is known to be a strong predictor of postoperative VTE in the broader literature, we repeated the multivariable analysis with this variable forced into the model and found similar results for the seven inception variables and *p* = 0.0540 for history of any VTE (OR 1.527, 95%CI 0.993–2.348), confirming stability of the model. Abbreviations: COPD—chronic obstructive pulmonary disease; CVA—cerebrovascular accident; Hx—personal history; LOS—length of stay; PTA—prior to admission; PVD—peripheral vascular disease; THA—total hip arthroplasty; TJA—total joint arthroplasty (of the hip or knee); TKA—total knee arthroplasty; VTE—venous thromboembolism (any).

Sixteen variables were found to be significantly different between the study groups in the univariable analysis (Table 4), of which eleven are captured by our current institutional risk stratification. After adjusting for multicollinearity, the final multivariable logistic regression model retained seven of these as being significant predictors of the composite complication primary outcome (Figure 4), of which four are included in our current stratification.

**Figure 5:**
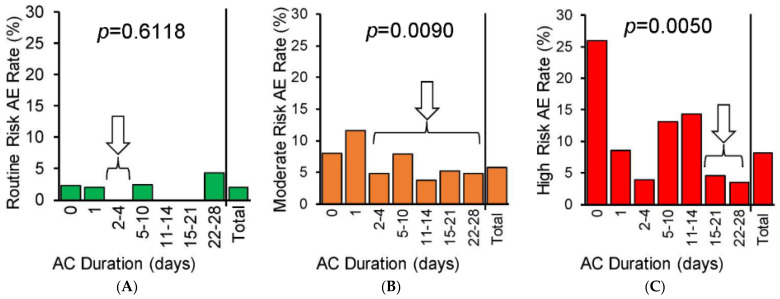
Composite complication primary outcome rate as a function of anticoagulant duration by VTE risk subgroup. Routine Risk (**A**, green bars, total n = 1074), Moderate Risk (**B**, orange bars, total n = 3744), and High Risk (**C**, red bars, total n = 295). Legend: anticoagulant duration of 0 days indicates an aspirin monotherapy chemoprophylaxis regimen. Arrows indicate expected nadir of antithrombotic-related complication risk for each subgroup. *p*-values for within-group chi-square tests overlaid on each panel, and Cochran–Mantel–Haenszel test *p* = 0.0006 for overall effect of VTE risk strata on the complication rate vs. anticoagulant chemoprophylaxis duration relationship. Only patients receiving 0–28-day anticoagulant chemoprophylaxis durations were included in these subgroup analyses to align with typical prescribing pattern timeframes, for conciseness. Abbreviations: AC—anticoagulant; AE—adverse event (e.g., ≥1 of the antithrombotic-related complications included in the primary outcome).

The complication rate as a function of anticoagulant prophylaxis duration for the three VTE risk subgroups is summarized in Figure 5. Patients at Routine risk had an overall 2% complication rate, with 0–2.4% rates observed among patients prescribed short anticoagulant durations but a 4.3% complication rate among those prescribed over three weeks of anticoagulant prophylaxis, though this distribution was not statistically significant (Figure 5A, *p* = 0.6118). Moderate risk patients saw an overall 5.7% complication rate and wider spread in the anticoagulant durations with lower complication rates (Figure 5B, *p* = 0.0090). High VTE Risk patients had the highest overall complication rate at 8.1%, which was found to be lowest at 3.4% with anticoagulant durations of 22–28 days. High VTE Risk patients who were prescribed aspirin monotherapy saw the highest complication rate at 25.9% (Figure 5C, *p* = 0.0050). The overall Cochran–Mantel–Haenszel test demonstrated a significant effect of VTE risk strata on the complication rate as a function of anticoagulant duration (*p* = 0.0006).

## 4. Discussion

Many present challenges complicate the selection of VTE chemoprophylaxis after elective TJA in modern enhanced recovery practice, and a patient-centered approach that limits the risks of both thrombotic and bleeding/wound complications is recommended [3,4,5,16,17,31,32]. When both categories are considered, antithrombotic-related complications were found to occur in approximately one of every 20 elective TJA patients in our study.

The duration of initial anticoagulant prophylaxis was not found to be a statistically significant predictor of the composite complication rate in our primary case–control analysis, with both Complication and No-Complication cohorts seeing a median duration of 5 days prior to aspirin (Figure 3). We expect this to be due to the various risk-benefit ratios expected with VTE prophylaxis strategies across different patient risk strata, and delineating the subgroup of patients most likely to benefit from longer anticoagulant exposures remains an important challenge. In the associated subgroup analysis exploring this further (Figure 3), we see more clear separation of the anticoagulant duration in the Moderate and High VTE Risk subgroups, though these were likely underpowered to see statistically significant differences.

Our a priori secondary analyses in Figure 5 explore the composite complication rate as a function of anticoagulant exposure within each VTE risk subgroup. While these should be viewed as exploratory based on the limitations of our study design and no adjustment for multiple comparisons, we feel it is clinically meaningful to bedside providers to visualize the differences in this relationship across various patient risk strata. The overall Cochran–Mantel–Haenszel test was highly significant (*p* < 0.001), indicating that the anticoagulant exposure-to-complication rate relationship differed greatly across the studied subgroups. We feel this suggests our current institutionally defined Routine, Moderate, or High VTE Risk stratification appears useful in discriminating patients with different complication-rate–anticoagulant-exposure relationships.

Based on regression and subgroup analyses, the results support many of the risk factors utilized in our stratification scheme as well as our current preferred chemoprophylaxis agents (aspirin and apixaban over enoxaparin). Our current approach to determining anticoagulant prophylaxis duration before aspirin (Figure 1) largely aligns with the durations of minimized complication rates seen across these subgroups and should be prospectively evaluated.

### 4.1. Contextualization with Prior Literature

Our institutional process for postoperative VTE prevention is robust in being multimodal, collaborative, and evidence-based and minimizes the limitations of prior risk models. We utilize a hybrid anticoagulant-to-aspirin chemoprophylaxis strategy to complete 4-week courses for higher risk patients, a rational approach based on the pathophysiology and time course of postoperative VTE [1,26,33,34] and validated by clinical trials as being comparably efficacious as longer anticoagulant-only courses [8,22]. This hybrid approach is melded with a modern evidence-based risk stratification aimed at tailoring anticoagulant duration to VTE risk (Figure 1). The majority of risk factors utilized in our current stratification scheme continue to be supported by recent evaluations [35], though further refinements may be indicated before prospective validation. Specifically, given the powerful influence of revision vs. primary surgery on the odds of antithrombotic-related complications seen here, separate risk stratification schemes may need to be developed.

Previous explorations of risk-stratified approaches to post-TJA VTE chemoprophylaxis have yielded encouraging results [11,12,13,14], though without the strength of a 3-level stratification scheme, as assessed in our study. Nearly three-quarters of our TJA population fell into a Moderate VTE Risk category, and this subgroup exhibited complication rates and a complication–anticoagulant exposure relationship distinct from the Routine or High VTE Risk subgroups (Figure 5). Simply dichotomizing patients into “Low Risk” vs. “High Risk” for VTE [13,36] may therefore limit both the sensitivity and specificity of a risk-stratified chemoprophylaxis strategy, potentially resulting in overtreating some patients and undertreating others [37]. Alternatively, complex VTE risk scoring tools such as the TJA-validated Caprini stratification may require significant resources to be effective in mitigating complications in clinical practice [10,17]. Our approach simplifies bedside risk assessment and chemoprophylaxis prescribing while individualizing complication prevention but needs prospective external validation before it can be recommended.

Aspirin has long been utilized as a more accessible alternative to potent anticoagulants that may have comparable safety and effectiveness, though selection bias in available studies is a concern and its routine use as monotherapy in higher risk patients remains controversial [20,38,39,40]. A large retrospective database analysis with propensity score matching recently suggested aspirin to be the safest chemoprophylaxis strategy after TJA across all patient risk strata, though selection bias, unmeasured confounding, and other limitations challenge its interpretation [36,37]. The newer direct oral anticoagulants, such as apixaban used in our practice, may offer reduced risk of VTE compared with aspirin without increasing bleeding/wound complications, as seen with earlier agents [41], though they can be costly. We address any cost barriers to apixaban through manufacturer coupons applied to electronic prescriptions prior to discharge.

Our study found that Routine Risk patients had low complication rates with very short courses of anticoagulants or aspirin alone, and lengthy anticoagulant courses may be associated with higher complication rates in this subgroup. Most patients were classified as Moderate VTE Risk per our institutional stratification and their optimal duration of anticoagulant before aspirin is less clear, possibly ranging from a few days to a few weeks. Lastly, heavily aspirin-based strategies appeared detrimental to the High VTE Risk subgroup, which comprised only 6% of the study population. This finding is corroborated by a subgroup analysis of a recent clinical trial in elective TJA in which high VTE Risk patients (those with personal history of VTE) experienced symptomatic VTE at an 8.5% rate with aspirin monotherapy as compared to 2.6% with enoxaparin chemoprophylaxis, while the interventions yielded more similar effects in the total study population [6]. These findings underscore the importance of tailoring anticoagulation strategies to specific risk profiles.

### 4.2. Limitations and Strengths

Our study is inherently limited in its single-center, retrospective, case–control design in terms of focused generalizability, susceptibility to selection bias and unmeasured or residual confounding, and potential for insufficient practice variability to construct an adequate counterfactual in all treatment exposures. Adherence to potentially complex prescribed chemoprophylaxis regimens, such as the actual timing of the patient deescalating from apixaban to aspirin, could not be assessed without patient contact, and other process improvements over time could not be adequately controlled for. Though these factors limit the internal validity of our study, our data still provide valuable insight into approximate “real world” complication rates associated with various prescribing strategies. While we used a multi-pronged approach to improve the capture of postoperative complications, our data collection was limited to those treated within our health system and so complication rates may still be underestimated. Specifically, minor complications may have been more likely to be treated at smaller outlying sites and not captured in our dataset. While a longer follow-up period would have been ideal for adjudicating the complication primary outcome, a 30-day follow-up is still clinically relevant since the vast majority of included events occur within the first 30 days after TJA [5,26,34]. A longer follow-up of 6 months would be optimal to observe delayed complications and secondary effects of the adopted prophylactic strategies.

Our regression model is further limited by sample size, residual confounding, and unmeasured confounding in that we could not capture many important risk factors retrospectively (e.g., smoking status, intraoperative details, postoperative mobility progression). These are important components of the complex dynamics of postoperative VTE that could not be accounted for, and this likely explains the wide and variable distribution of complication rates observed in the Moderate VTE Risk subgroup to some degree. Additionally, some risk factors were not prevalent enough to reach significant associations, though they are known to confer VTE risk based on the broader literature base (e.g., personal history of VTE), likely due to issues with retrospective data queries based on diagnosis codes and suboptimal sample size. The reliance on diagnosis codes for collecting risk factor data collection could further introduce biases whereby patients with less frequent interactions with the healthcare system, and/or those who completed pre-admission testing outside the organization, had fewer pre-existing diagnoses codified and were classified as lower risk for VTE in our database. This could reduce the accuracy and precision of the regression model and secondary analyses of the complication rate-anticoagulant duration relationship. Ideally, our study would have built three risk prediction models, one for each risk stratum, but the small sizes of subgroups precluded this.

This study has several strengths. We assessed a risk-stratified approach to VTE chemoprophylaxis, including both bleeding and thrombotic complications, addressing a key patient-centered research priority [5]. While such a composite primary outcome is a departure from VTE-based outcomes used in prior studies in TJA, we felt it was valuable and novel to account for both thrombotic and bleeding/wound complications when assessing a VTE chemoprophylaxis strategy. We were also careful to include only clinically important thrombotic events in the composite primary outcome and assessed patients in all risk categories. Using our data to inform design, future studies could better explore antithrombotic-related complications’ rates associated with various VTE chemoprophylaxis approaches via larger database analyses, and ultimately prospectively validate a refined risk-stratified prescribing tool.

## 5. Conclusions

Our single-center experience adds to current literature assessing patient-specific approaches to VTE chemoprophylaxis after elective TJA. When both bleeding and thrombotic adverse events are considered in the first 30 postoperative days, the use of initial anticoagulant prophylaxis prior to aspirin was associated with lower rates of antithrombotic-related complications than an aspirin-only approach in higher risk patients, while patients at routine VTE risk saw no apparent benefit over aspirin monotherapy. Our practical and collaborative approach to risk-stratified VTE chemoprophylaxis should be refined and prospectively explored as a tool for limiting antithrombotic-related complications after elective TJA.

## Figures and Tables

**Table 1:** Antithrombotic-related complication composite primary outcome definition.

Thrombotic Adverse Events	Hemorrhagic/Wound Adverse Events
Acute DVT, excluding isolated distal DVT Acute PE, excluding isolated subsegmental PE Acute MI, with or without ST-segment elevation Acute ischemic CVA Acute systemic arterial thrombosis	Readmission for wound complication * or for bleeding at any site, including any bleed- or anemia-related admitting diagnosis Reoperation for wound complication * or bleeding Transfusion of 2 or more units PRBCs for any indication

* Includes wound hematoma/seroma, wound infections, and other disruptions of the wound or superficial tissues post-procedure. Legend: the primary outcome included any occurrence of one or more listed complications within 30 days of surgery. CVA—cerebrovascular accident; DVT—deep vein thrombosis; MI—myocardial infarction; PE—pulmonary embolism; PRBCs—packed red blood cells.

**Table 2:** Antithrombotic-related complications composite primary outcome and component rates.

Variable	N	% of Total Population N = 5405	% of Complication Population n = 279
Any VTE	48	0.9	17.2
DVT	38	0.7	13.6
PE	10	0.2	3.6
Any arterial thrombotic event	23	0.4	8.2
CVA	3	0.06	1.1
MI	4	0.07	1.4
Other arterial thrombosis	17	0.3	6.1
Readmission for wound complication or any-site bleeding	82	1.5	29.4
Reoperation for wound complication or bleeding	82	1.5	29.4
Blood transfusion 2+ units PRBCs	106	2	38

Footnote: a total of 5405 patients were analyzed, 279 (5.2%) of which experienced an antithrombotic-related adverse event (the “Complication Population”). Abbreviations: CVA—ischemic cerebrovascular accident; DVT—deep vein thrombosis (isolated distal DVTs not included); MI—myocardial infarction (includes ST elevated and non-ST elevated MIs); PE—pulmonary embolism (isolated subsegmental PEs not included); PRBCs—packed red blood cells.

**Table 3:** Antithrombotic-related complications’ composite primary outcome rate by patient VTE risk strata and anticoagulation exposure subgroups.

	Total Population N = 5405	Composite Complication Primary Outcome		
		No n = 5126	Yes n = 279	*p*-Value
**VTE Risk Strata, n (%)**				<0.001
Routine	1084 (20)	1062 (20.7)	22 (7.9)	
Moderate	3993 (74)	3764 (73.4)	229 (82.1)	
High	327 (6)	300 (5.9)	27 (9.7)	
Exposure Groups *, n (%)				0.0003
Aspirin Only	639 (11.8)	605 (11.8)	34 (12.2)	
Enoxaparin then Aspirin	3207 (59.3)	3011 (58.7)	196 (70.3)	
Apixaban then Aspirin	1461 (27)	1416 (27.6)	45 (16.1)	

Footnote: a significant differential distribution of the primary outcome was observed across the VTE chemoprophylaxis exposure subgroups and across our institutionally defined VTE risk strata. Bolded *p*-values indicate statistically significant findings at prespecified alpha = 0.05. *: Remaining patients were initially started on one anticoagulant but switched to the other, prior to de-escalation to aspirin (usually only carried out when needed to ensure insurance coverage). Abbreviation: VT—venous thromboembolism.

**Table 4:** Demographic data and univariable analysis.

	Total Population N = 5405	Composite Complication Primary Outcome		
		No n = 5126	Yes n = 279	*p*-Value
**Demographics**				
Age, mean (SD)	62.1 (10.2)	62 (56–68)	63 (54–73)	0.1773
Age ≥ 80, n (%)	247 (4.6)	227 (4.4)	20 (8.7)	**0.0328**
Sex, n (%)				0.3740
Female	3141 (58.1)	2986 (58.3)	155 (55.6)	
Male	2264 (41.9)	2140 (41.7)	124 (44.4)	
Weight (kg), mean (SD)	92.8 (20.1)	92.8 (20)	93.7 (22.2)	0.4644
BMI (kg/m^2^), median (IQR)	32 (28–36)	32 (28–36)	32.1 (27–36)	0.8407
BMI 35–39 (kg/m^2^), n (%)	1163 (21.5)	1101 (21.5)	62 (22.2)	0.7685
BMI ≥ 40 (kg/m^2^), n (%)	510 (9.4)	478 (9.3)	32 (11.5)	0.2327
**Medical History, n (%)**				
Any VTE	310 (5.7)	284 (5.5)	26 (9.3)	**0.0082**
DVT	309 (5.7)	283 (5.5)	26 (9.3)	**0.0078**
PE	1 (<0.1)	1 (<0.1)	0 (0)	1.0000
Hypercoagulable Disorder	44 (0.8)	41 (0.8)	3 (1)	0.4946
Active Cancer	2 (<0.1)	2 (<0.1)	0 (0)	1.0000
CAD/Ischemic Disease	468 (8.7)	428 (8.3)	40 (14.3)	**0.0005**
Heart Failure	124 (2.3)	110 (2.1)	14 (5)	**0.0018**
Atrial Fibrillation	150 (2.8)	137 (2.6)	13 (4.7)	**0.0491**
Cardiomyopathy	50 (0.9)	47 (0.9)	3 (1.1)	0.7428
Valvular Disease	48 (0.9)	43 (0.8)	5 (1.8)	0.0996
Varicose Veins	4 (<0.1)	3 (<0.1)	1 (0.4)	0.1911
PVD/Atherosclerosis	113 (2.1)	98 (1.9)	15 (5.4)	**<0.0001**
COPD	271 (5)	353 (6.9)	36 (12.9)	**<0.0001**
OSA	1009 (18.7)	937 (18.3)	72 (25.8)	**0.0017**
Ischemic CVA	274 (5)	247 (4.8)	27 (9.7)	**0.0003**
Tobacco Use	832 (15.4)	776 (15.1)	56 (20)	**0.0262**
Hemophilia	5 (<0.1)	5 (<0.1)	0 (0)	1.0000
On Aspirin PTA	300 (5.5)	272 (5.3)	28 (10)	**0.0008**
On Hormonal Agent PTA	57 (1.1)	56 (1.1)	1 (0.4)	0.3679
**Procedure/Hospitalization Data**				
Operative Joint, n (%)				**0.0129**
THA	1967 (36.4)	1846 (36)	121 (43.4)	
TKA	3438 (63.6)	3280 (64)	158 (56.6)	
Procedure Type, n (%)				**<0.0001**
Primary	4837 (89.5)	4632 (90.4)	205 (73.5)	
Revision	568 (10.5)	494 (9.6)	74 (26.5)	
LOS (days), median (IQR)	3.4 (3.1–4.3)	3.4 (3.1–4.3)	4.4 (3.4–5.5)	**<0.0001**
LOS > 3 days, n (%).	4133 (76.5)	3881 (75.7)	252 (90.3)	**<0.0001**

Footnote: bolded *p*-values indicate statistically significant findings at prespecified alpha = 0.05. Abbreviations: BMI—body mass index; CAD—coronary artery disease; COPD—chronic obstructive pulmonary disease; CVA—cerebrovascular accident; DVT—deep venous thrombosis (any); IQR—interquartile range; LOS—length of stay; OSA—obstructive sleep apnea; PE—pulmonary embolism (any); PTA—prior to admission; PVD—peripheral vascular disease; SD—standard deviation; THA—total hip arthroplasty; TKA—total knee arthroplasty; VTE—venous thromboembolism.

## Data Availability

The data presented in this study are available on request from the corresponding author.

## Supplementary Materials

The following supporting information can be downloaded at: https://www.mdpi.com/article/10.3390/jcm14020366/s1, Table S1: Diagnosis codes utilized.
